# A Machine Learning Model for Predicting the Propagation Rate Coefficient in Free-Radical Polymerization

**DOI:** 10.3390/molecules29194694

**Published:** 2024-10-03

**Authors:** Yiming Wang, Yue Fang, Haifan Zhou, Hanyu Gao

**Affiliations:** Department of Chemical and Biological Engineering, The Hong Kong University of Science and Technology, Hong Kong 999077, China; ywangsx@connect.ust.hk (Y.W.); yfangax@connect.ust.hk (Y.F.); hzhoubz@connect.ust.hk (H.Z.)

**Keywords:** propagation rate coefficient, SMILES, lasso regression, Molecular Transformer embeddings

## Abstract

The propagation rate coefficient (*k*_p_) is one of the most crucial kinetic parameters in free-radical polymerization (FRP) as it directly governs the rate of polymerization and the resulting molecular weight distribution. The *k*_p_ in FRP can typically be obtained through experimental measurements or quantum chemical calculations, both of which can be time consuming and resource intensive. Herein, we developed a machine learning model based solely on the structural features of monomers involved in FRP, utilizing molecular embedding and a Lasso regression algorithm to predict *k*_p_ more efficiently and accurately. The result shows that the model achieves a mean absolute percentage error (MAPE) of only 5.49% in the predictions for four new monomers, which indicates that the model exhibits strong generalization capabilities and provides reliable and robust predictions. In addition, this model can accurately predict the influence of the ester side chain length of (meth)acrylates on *k*_p_, aligning well with established scientific knowledge. This approach offers a straightforward and practical model for other researchers to rapidly obtain accurate *k*_p_ values by employing monomer structural information. The model is sufficiently general to apply to a wide range of (meth)acrylate and butadiene FRP monomers, thereby supporting kinetic modeling of polymerization reactions.

## 1. Introduction

Kinetic rate coefficients in free-radical polymerization (FRP) are crucial in polymerization modeling and optimization, which can lead to the design and synthesis of novel materials [1]. These parameters are an integral part of a microkinetic model of polymerization (including chemical reaction mechanisms at the molecular level and differential equations describing concentration changes over time), which can predict important reaction performance metrics including monomer conversion, molecular weight distribution, and dispersity. Among these key kinetic parameters, the propagation rate coefficient (*k*_p_) holds particular significance since *k*_p_ governs the overall rate of the polymerization reaction and reflects the inherent reactivity of the monomer so that it can be used to model the polymerization behavior of different monomers [2].

The *k*_p_ values are typically obtained through a variety of approaches, including direct and indirect experimental measurements as well as quantum chemical calculations [3,4,5]. Direct experimental measurements of the *k*_p_ are typically conducted via the pulsed laser polymerization–size exclusion chromatography (PLP-SEC) technique, based on the rapid and periodic formation of primary radicals induced by high-frequency laser pulses [6,7]. Kinetic modeling and regression analysis serve as indirect experimental methods to predict *k*_p_ from measured temporal change of concentration data [8]. In addition to these experimental approaches, quantum chemical calculations employing high-level ab initio molecular orbital theory have been utilized to provide mechanistic insights and estimate *k*_p_ values [3].

However, these methods have their own limitations. For the direct experimental method, PLP-SEC is the standard method recommended by the IUPAC Working Party for the experimental determination of kinetic parameters such as the *k*_p_, activation energy (*E*_A_), and pre-exponential factor (*A*) in FRP [9]. This technique employs laser pulses to periodically induce radical formation in monomer solutions, resulting in characteristic peaks in the polymer molecular weight distribution that enable the determination of the *k*_p_. By measuring *k*_p_ at multiple temperatures using this approach, the Arrhenius equation for the propagation of that monomer can be derived. Guided by this principle, Buback et al. have determined the Arrhenius equation for the *k*_p_ of styrene, while Beuermann et al. have carried out similar work for methyl methacrylate, ethyl methacrylate, and dodecyl methacrylate [7,10,11]. However, these measurements can be quite complex and time consuming, and reliable *k*_p_ values require IUPAC harmonization of data submitted from different laboratories. Kinetic modeling and regression analysis are indirect experimental approaches to computationally determine the *k*_p_ in FRP, including deterministic methods and stochastic approaches. For instance, Zhou et al. utilized the method of moments to model the apparent *k*_p_ for the polymerization of butyl methacrylate and methyl methacrylate at 25 °C [5], and the results were found to be consistent with PLP-SEC data. Marien et al. developed an isothermal kinetic Monte Carlo model that accounts for all relevant elementary reactions to accurately simulate the complete PLP-SEC traces and extract the *k*_p_ for n-butyl acrylate [8]. However, this is essentially a data-fitting process that still relies on experimental measurements of the dynamic changes of concentrations, which lacks predictive power. Apart from these experimental methods, quantum chemical calculations coupled with transition state theory and high-level ab initio molecular orbital theory have been employed to calculate intrinsic rate coefficients of FRP. For example, Heuts et al. utilized quantum chemical calculations to determine the geometries, energies of reactants, vibrational frequencies, and the transition state, and then applied transition state theory to obtain the Arrhenius parameters [3]. The calculated results for ethylene propagation were in good agreement with PLP-SEC data, although this approach is limited to propagation reactions that are not significantly influenced by the presence of solvents. Huang et al. calculated the *k*_p_ values for acrylonitrile and methacrylonitrile, and investigated the hindering effect of the methyl substituent on the rotational degrees of freedom in the transition state through quantum chemical calculations [12]. However, the accuracy of *k*_p_ values obtained from quantum chemical calculations depends on the precise description of the energetic and entropic barriers, and as the number of atoms in the monomer molecules increases, the computational cost and time required would increase dramatically.

Recently, researchers have turned to the use of machine learning methods to predict *k_p_* values. Reydt et al. achieved good fitting results using machine learning for (meth)acrylate-type FRP monomers [13]. They utilized GAMESS and ChemSpider to obtain various physicochemical properties of the monomers, including dipole moment, boiling point, melting point, surface tension, refractive index, and polarizability, and used these as features in a ridge regression model to predict *k*_p_. While the model showed reasonable predictive performance for (meth)acrylate monomers, its ability to predict kinetic parameters for other FRP monomers, such as butadiene-type and styrene-type, was limited. Furthermore, the complexity of their features, which required computational software (GAMESS, version: 2018, R1) to obtain, also restricted the generalization capability of their model in predicting *k*_p_ for new monomers. Recently, Shi et al. developed a quantitative structure–property relationship model based on density functional theory and machine learning regression analysis to predict Arrhenius parameters and *k*_p_, achieving high accuracy [14]. However, this machine learning approach still requires complex quantum chemical calculations, which are time consuming. Therefore, it is desirable to develop a generalized, accurate, and computationally efficient machine learning model for the prediction of *k*_p_.

Herein, we propose a machine learning model based on the molecular structure of monomers to predict *k*_p_ values and Arrhenius parameters. Firstly, the PubChem database was utilized to obtain the Simplified Molecular-Input Line-Entry System (SMILES) representations of various monomers [15]. Subsequently, the SMILES was converted into both Molecular ACCess System (MACCS) fingerprints and Molecular Transformer embeddings [16,17], and these two types of fingerprints were then combined as the input features. The features were used to predict *k*_p_ using a Lasso regression model with a regularization term [18], which enabled automated feature selection of more influential variables while also preventing overfitting. To validate the generalizability of our model, monomers from external datasets were tested using this model. We demonstrated that by simply using molecular fingerprints derived from 2D molecular graphs (or the equivalent SMILES representation), the propagation rate coefficients can be predicted for (meth)acrylate and butadiene monomers with high accuracy, which can strongly contribute to the simulation of polymerization reactions and design of polymerization systems.

## 2. Results and Discussion

The fitting performance of the four regression models was first evaluated on the training set of 41 monomers under the same standards as shown in Table S1. Furthermore, the predictive capability of the trained models was compared on monomers outside the training set to select the models with the strongest generalization ability. Finally, reasonable predictions were also made for the Arrhenius pre-exponential factor *A* and activation energy *E*_A_ to predict the *k*_p_ values of new monomers at different temperatures.

### 2.1. Comparison of Four Regression Models on the Training Dataset

The use of ln(*k*_p_) values at 25 °C was adopted for convenient comparison across the different models. The use of the natural logarithm of the rate constant, rather than the raw *k*_p_ values, was employed to avoid the models predicting unphysical negative values. This approach also enhanced the statistical validity of the regression analysis, resulting in more robust and reliable models.

**Figure 1:**
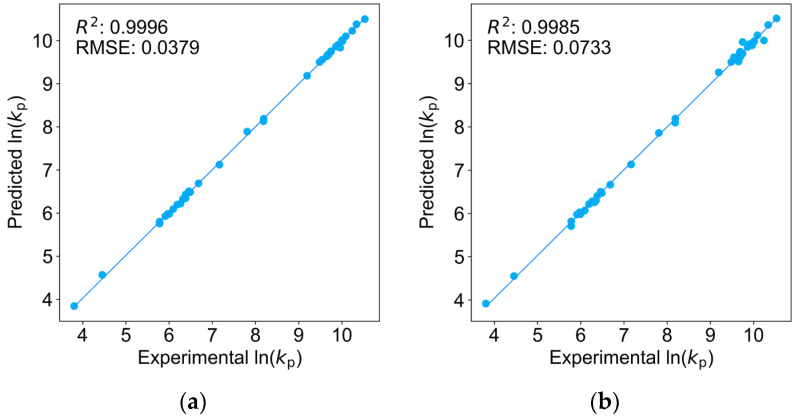

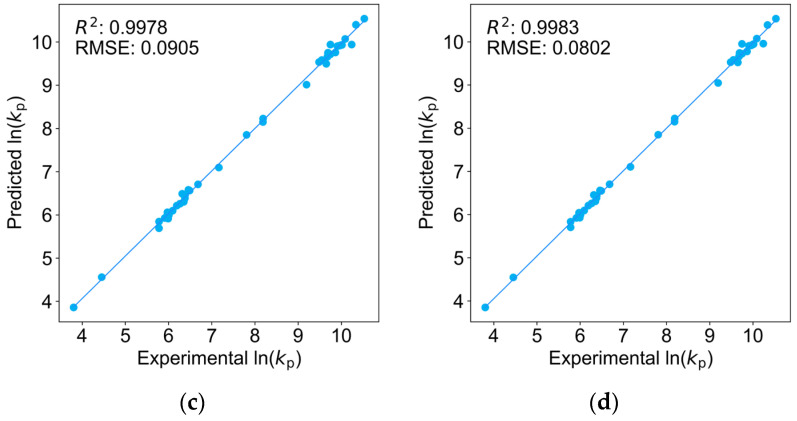
Results of the fitting analyses for predicting ln(*k*_p_)^25°C^ versus experimental ln(*k*_p_)^25°C^: (**a**) multivariate linear regression model; (**b**) Lasso regression model; (**c**) ridge regression model; (**d**) Bayesian ridge regression model [13].

As shown in Figure 1, the fitting results of the four regression models all exhibited high quality, with coefficient of determination (*R*^2^, Equation (S1)) values exceeding 0.9978 and root-mean-square errors (RMSE, Equation (S2)) less than 0.1000. This demonstrates the excellent fitting performance of these regression models on the training dataset and also indicates that the feature transformation process effectively preserved the majority of the structural information.

**Figure 2:**
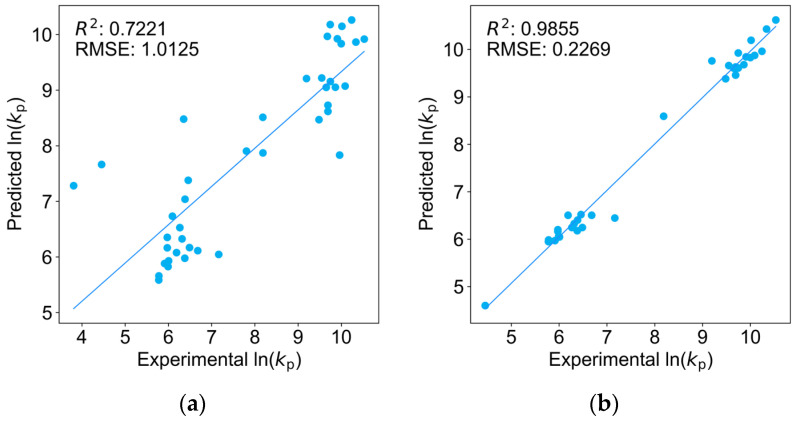
Regression models trained by Reydt et al. (**a**) All monomers (*R*^2^ = 0.7221, RMSE = 1.0125); (**b**) (meth)acrylates, styrene, and acrylonitrile (*R*^2^ = 0.9855, RMSE = 0.2269) [13].

Figure 2 presents the fitting performance using the features selected by Reydt et al. [13], including molecular weight, polarity, and boiling point. Figure 2a shows the results for the same 41 monomers, while Figure 2b displays their fitting on a training set comprising (meth)acrylates, styrene, and acrylonitrile, after excluding monomers that could not be adequately described by their feature set. In the latter case, their model achieved an *R*^2^ of 0.9855 and an RMSE of 0.2269, still inferior to any of the four regression models presented here.

To provide a more intuitive comparison of the prediction errors across the four models, the absolute percentage error (APE, Equation (S3)) between the predicted and actual *k*_p_ values was also examined.

**Figure 3:**
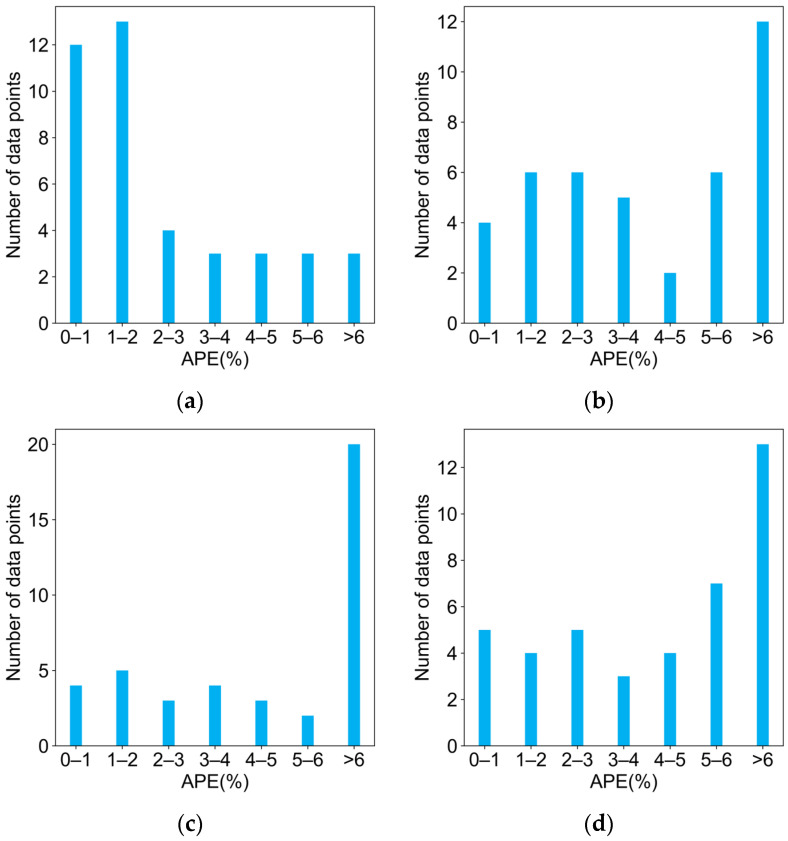
APE distribution of predicted *k*_p_^25 °C^ and experimental *k*_p_^25 °C^ for (**a**) multivariate linear regression; (**b**) Lasso regression; (**c**) ridge regression; and (**d**) Bayesian ridge regression.

As depicted in Figure 3, consistent with the results evaluated using *R*^2^ or RMSE, the multivariate linear regression model exhibited the most favorable performance, with over 70% of the monomers having APEs below 3%, further corroborating the efficacy of the selected features. In contrast, the ridge regression model showed the poorest performance, with nearly 50% of the monomers having APEs greater than 6%, indicating that the addition of the regularization penalty in ridge regression was not well suited for this training set. The Bayesian ridge regression, which combined the characteristics of ridge and Lasso regressions, yielded slightly inferior fitting results on the training set compared to Lasso regression, suggesting that the regularization term in Lasso regression was better able to capture the inherent relationship between the features and ln(*k*_p_). However, given the extremely limited data points, the superior performance on the training set may be attributed to overfitting, which could lead to poor predictive capabilities for new monomers. Therefore, the four models were evaluated on a separate test dataset of new monomers.

### 2.2. Construction of the External Test Dataset

In order to test the generalizability of the model, we obtained a few more data points from the literature outside the training set. As shown in Table 1, the kinetic data for dodecyl acrylate, tridecyl acrylate, tert-butyl methacrylate, and chloroprene were obtained from four different experimental laboratories to reduce the overall impact of experimental errors associated with PLP-SEC measurements on the evaluation of the model [19,20,21,22]. Notably, the data reported for tridecyl acrylate were actually obtained from a sample of Tridecyl N acrylate, which is a combination of isomers of tridecyl acrylate with partially esterified side chains [20]. In contrast, the literature reported that tridecyl A acrylate represents a distinct set of tridecyl acrylate isomers. Nevertheless, the *k*_p_ value of tridecyl N acrylate is larger than that of dodecyl acrylate, while the *k*_p_ value of tridecyl A acrylate is smaller than that of dodecyl acrylate. Based on the general trend that longer acrylate ester side chains lead to larger *k*_p_ values [4], we inferred that the tridecyl N acrylate monomer had a relatively lower degree of side chain branching, and thus considered its *k*_p_ value as a reference for tridecyl acrylate (TDA). Furthermore, the rationality of the model predictions was then interpreted using the scientific principles of FRP.

### 2.3. Comparison of Four Regression Models on the Test Dataset

**Figure 4:**
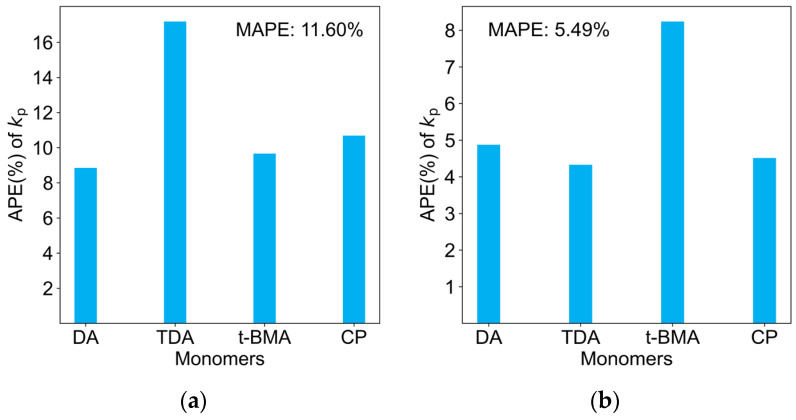

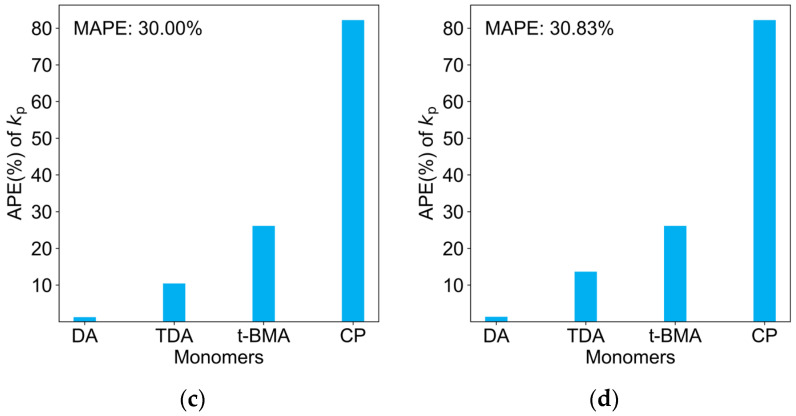
Predictive results on the test dataset: (**a**) multivariate linear regression; (**b**) Lasso regression; (**c**) ridge regression; (**d**) Bayesian ridge regression.

As shown in Figure 4, mean absolute percentage error (MAPE, Equation (S4)) was used to evaluate the overall predictive capability of the models on the four new monomers, while the individual APE values for each of the four monomers could detect the predictive capability of the models on different types of monomers.

As depicted in Figure 4c,d, not only did ridge regression and Bayesian ridge regression exhibit poor performance on the training set, but they also showed relatively inferior performance on the four new monomers, with MAPE values of 30.00% and 30.83%, respectively. The rationale behind this is that the regularization method employed in ridge regression is more appropriate for situations characterized by multicollinearity among the predictor variables. Conversely, if the feature set comprises orthogonal predictor variables, ridge regression may not demonstrate optimal performance. This suggests that the sub-structural features of monomers have independent impacts on the final *k*_p_ values in FRP.

Additionally, the simplest direct multivariate linear regression model also exhibited acceptable results, with a MAPE of only 11.60% on the test dataset. This indicates that the features we inputted were sufficient to represent the monomer sub-structures that influence the chain propagation rate. However, the multivariate linear regression model failed to correctly validate the scientific principle that longer acrylate ester side chains lead to larger *k*_p_ values [4]. For example, at 25 °C, the predicted *k*_p_ values for ethyl acrylate and propyl acrylate were 21,713 and 13,007 L mol^−1^ s^−1^, respectively. This suggests a lack of a regularization penalty, which may have caused a certain degree of overfitting on the training set.

Lasso regression showed the best test results, with a MAPE of only 5.49% for the four new monomers, and each monomer’s prediction bias was less than 8%. The rationale is that Lasso regression minimizes the sum of the absolute values of the regression coefficients, enabling it to shrink the coefficients of secondary variables to exactly zero while retaining the primary variables that influence the *k*_p_ values. Consequently, during the training process, the Lasso regression model can automatically select the key sub-structural features of the monomers that impact the *k*_p_ values, such as methyl substitutions on carbon–carbon double bonds, halogen substituents, and ester side chain lengths.

Overall, with the SMILES representations of the monomers as the initial features and proper feature engineering, Lasso regression outperforms any current ab initio calculations and the machine learning model of Reydt et al. [13], which used partial monomer properties as features, in predicting *k*_p_.

### 2.4. Reflection of Scientific Principles

In the field of FRP, two well-established scientific laws have been validated. The first law states that for linear alkyl (meth)acrylates, the *k*_p_ gradually increases as the number of carbon atoms in the ester side chain increases [4,20]. The second law suggests that for (meth)acrylate esters with the same side chain, the *k*_p_ of the acrylate ester is two orders of magnitude higher than that of the corresponding methacrylate ester [13]. To check against these two postulates, the optimal Lasso regression model was employed to predict the *k*_p_ values of several new monomers, and the results are presented in Table 2.

The predictive results show that as the number of side-chain carbon atoms increases from 12 to 15 for dodecyl acrylate, tridecyl acrylate, tetradecyl acrylate, and pentadecyl acrylate, the *k*_p_ values indeed gradually increase from 17,682 to 22,635. Furthermore, the *k*_p_ value of Tetradecyl acrylate (22,034) is two orders of magnitude higher than that of Tetradecyl methacrylate (611), in accordance with the established scientific law. The predictive results that align with these scientific laws demonstrate the significant potential of our model in accurately and reliably predicting the *k*_p_ values of new monomers.

### 2.5. Predictions of k_p_ at Multiple Temperatures, A, and E_A_

**Figure 5:**
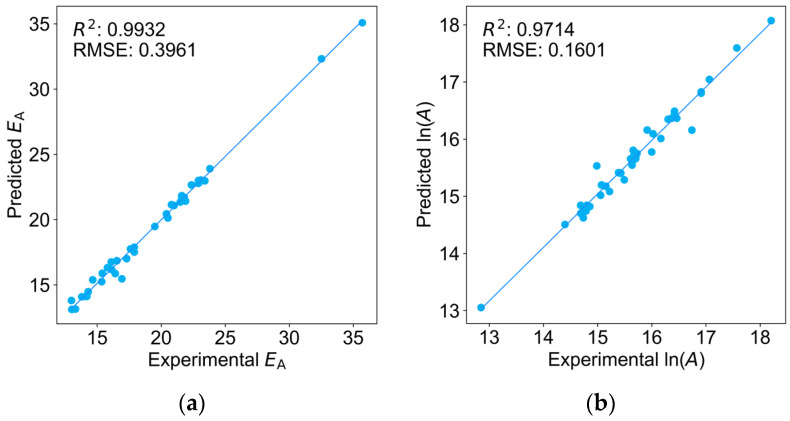
Fitting analyses on the training set for (**a**) *E*_A_; (**b**) ln(*A*).

As in the manner of obtaining the Arrhenius parameters from the PLP-SEC experiments, *k*_p_ values of monomers at different temperatures were simultaneously predicted and linearly fitted to yield the *A* and *E*_A_. Specifically, the *k*_p_ values at 15 °C, 25 °C, 35 °C, 45 °C, 55 °C, and 65 °C were used as labels to train six well-fitted models, as shown in Figure S9. The *R*^2^ values for these models were all above 0.9980, and the RMSEs were all below 0.0800. Subsequently, the *A* and *E*_A_ parameters were obtained by linearly fitting the *k*_p_ values at the six temperatures according to the Arrhenius equation, and the results of the training set are presented in Figure 5.

In contrast to the optimization of a single predictive model, the error associated with forecasting the parameters *A* and *E*_A_ using the ensemble of six models will exhibit a marginal increase. However, this is still far superior to the results reported by Reydt et al. [13]. Our *E*_A_ (*R*^2^ = 0.9932, RMSE = 0.3961) and ln(*A*) (*R*^2^ = 0.9714, RMSE = 0.1601) outperform their *E*_A_ (*R*^2^ = 0.9630, RMSE = 0.8230) and ln(*A*) (*R*^2^ = 0.6660, RMSE = 0.4270). This further demonstrates the effectiveness of our model in directly using SMILES structures for prediction.

As shown in Table 3, the predicted Arrhenius parameters *E*_A_ and *A* for the four new monomers on the test dataset are also provided. First, the six well-fitted models obtained from the training set were employed to predict the *k*_p_ values of the four new monomers at the six temperatures. Subsequently, the *E*_A_ and *A* parameters were derived by linearly fitting the predicted *k*_p_ values according to the Arrhenius equation.

**Figure 6:**
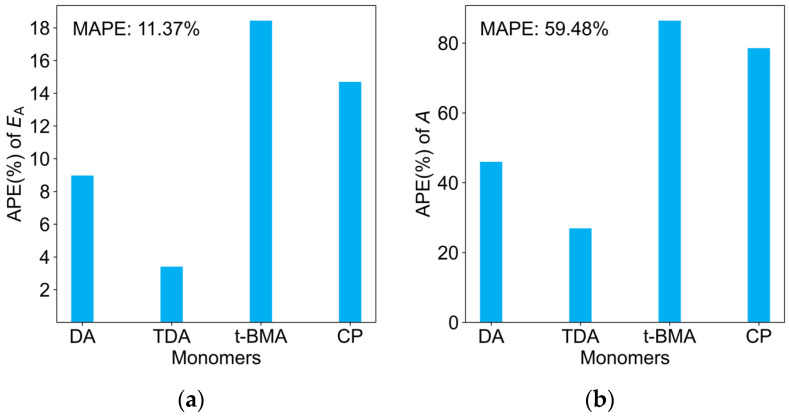
APE of predicted and experimental values on the test dataset for (**a**) *E*_A_; (**b**) *A*.

The errors between the predicted and experimental values are illustrated in Figure 6. For the *E*_A_ predictions, the MAPE for the four new monomers was only 11.37%, and the APE was below 18% for all monomers. This further demonstrates the high accuracy and robustness of the model in predicting *k*_p_ values. In contrast, the prediction error for the *A* values was relatively larger, with a MAPE of 59.48%.

However, it is important to note that the PLP-SEC experiments used to measure *k*_p_ values inherently have an experimental uncertainty of 10–15% [23]. While this error is orthogonal to model prediction errors (our model is trained to predict the single-point experimental *k*_p_ values without considering these experimental uncertainties), it is reasonable to conclude that an error level of 10–15% in *k*_p_ values is practically acceptable. The *A* values are derived from fits of *k*_p_ values across multiple temperatures, and in this fitting process, *A* values are very sensitive to small changes in *E*_A_ values. Considering this, the prediction error for the *A* parameter is deemed acceptable in the context of FRP modeling.

Although our model is capable of providing reliable *k*_p_ value predictions for new (meth)acrylate and butadiene FRP monomers, limitations still exist. The accuracy of the *k*_p_ predictions may decline when certain sub-structural features of monomers are absent from the training set. Therefore, to enhance the model’s generalization capability, it is essential to expand the dataset to include a broader variety of FRP monomers. Furthermore, due to the inherent uncertainty in the PLP-SEC experimental data, although a small APE is exhibited by the predicted results compared to the PLP-SEC data, the APE in relation to the objectively true *k*_p_ values may be larger.

## 3. Methods

### 3.1. Construction of the Training Dataset

The dataset of kinetic parameters for the monomers of FRP used to train our model was presented in Tables S1 and S2. These data have been curated by Reydt et al. [13]. The majority of the data originate from the benchmark datasets recognized by IUPAC, while the remaining data come from individual laboratory experiments that are also considered reliable under IUPAC standards. The *k*_p_ values at different temperatures were given by the Arrhenius equation:

(1) $$k_{p}=A\exp\left(-\frac{E_{A}}{RT}\right)$$

where *A* is the pre-exponential factor, *E*_A_ is the activation energy for propagation reactions, *R* is the universal gas constant, and *T* is the absolute temperature.

### 3.2. Feature Representation

#### 3.2.1. Scientific Understanding

Reydt et al. achieved satisfactory model fitting results in their development of machine learning models for the prediction of *k*_p_ by using intrinsic properties of the monomers as features, such as molecular weight, dipole moment, boiling point, melting point, and dissociation constant [13]. Specifically, molecular weight can indirectly influence *k*_p_ by reflecting changes in the length of the ester side chains in (meth)acrylate monomers. Dipole moment captures differences in polarity, which can directly impact both the reactivity in free-radical reactions and the solvation environment during bulk polymerization, thereby affecting the rate of propagation. The other features serve as complementary descriptors of the monomers’ molecular properties.

However, even though they identified the key molecular properties of monomers that influence the propagation rate, it remains challenging to provide a comprehensive representation of the molecular properties affecting *k*_p_. Other potentially influential molecular characteristics, such as the spatial environment of double bonds, may have been overlooked as features in the model due to the difficulty in their quantitative description. Furthermore, some properties affecting *k*_p_ that are not well understood could not be incorporated. In addition, when applying the model to predict the *k*_p_ of new monomers, the physical properties might be unknown (e.g., melting point), which limits the generalizability of this approach.

Since molecular properties are fundamentally determined by molecular structure, utilizing the complete molecular structure of the monomers as features for the machine learning model could resolve the issue of incomplete statistical representation of molecular properties. Herein, we used the SMILES representation of the monomer molecules as the initial features, which were then transformed into an encoding format suitable for machine learning to construct an accurate and generalizable model.

#### 3.2.2. SMILES and MACCS Fingerprints

The SMILES, based on the principles of molecular graph theory, provides a standardized representation of molecular structures, capturing information about atoms, bonds, aromaticity, stereochemistry, and other molecular features [15]. This makes the SMILES the raw input for our model. MACCS fingerprints and Molecular Transformer embeddings are two widely adopted methods for converting SMILES representations into molecular features. 

MACCS fingerprints are binary vectors of 166 bits, where each bit represents the presence (1) or absence (0) of a predefined structural fragment within the molecule [17]. However, while MACCS fingerprints capture the main atomic, bond, and functional group information of a molecule, using this fingerprint alone may not be sufficient to represent the complete structural details of FRP monomers. For instance, dodecyl acrylate and tridecyl acrylate have the same structure, except for a single carbon atom difference in the ester side chain. As shown in Table S4, a model using only MACCS fingerprints as input provided the same predicted results for these two monomers, indicating they possess completely identical sets of the 166 structural fragments and therefore have the same fingerprints.

#### 3.2.3. Molecular Transformer Embeddings

SMILES representations of monomers were simultaneously converted into Molecular Transformer embeddings as a complement to MACCS fingerprints, to maximize the retention of molecular structural information [16]. The Molecular Transformer is a deep learning model that can generate embeddings (vector representations) from the SMILES strings of molecules, capturing their structural information. It is based on the Transformer architecture, consisting of an encoder and a decoder, and here we utilize only the encoder to obtain the molecular embeddings.

The conversion process involves tokenization, positional encoding, an embedding layer, a Transformer encoder, layer normalization, and output aggregation. During these processes, the positional encoding and the attention mechanism of the Transformer itself play an important role in preserving the structural information of small molecules [24]. The positional encoding can reflect the relative positional information of the atoms in the SMILES representation, which better captures the information of the cyclic structure and the length of side chains of the molecules. Furthermore, the global perception ability brought by the attention mechanism allows each atom or functional group to focus on any other element in the SMILES sequence, which can better represent the resonance effects, conjugation effects, and the overall structure of the molecules.

However, reliable Molecular Transformer embeddings for molecular structures require extensive training to determine the optimal settings of the Transformer architecture parameters. As shown in Figure S1, Morris et al. collected 8,300,000 molecular SMILES strings and IUPAC names from PubChem to train the Transformer model [16]. Additionally, they have made the pre-trained Transformer models publicly available, which were subsequently utilized by us to obtain embeddings for the 41 FRP monomers in our dataset. The resulting embeddings are 2D matrices with the number of rows equal to the length of the SMILES strings and the number of columns set to 512. These matrices were then averaged across the rows to produce 1D matrices with 512 columns, which were used as the input for regression models.

Ultimately, the 1D vector MACCS fingerprints and the 1D matrix of the Molecular Transformer embeddings were concatenated as the feature inputs of the 41 monomers for regression models. As illustrated in Supplementary Figure S2, SMILES strings were respectively transformed into MACCS fingerprints, Molecular Transformer embeddings, and their combination encodings, which were then used to train three separate predictive models. The results (Figures S4–S6) demonstrate that integrating these two encoding approaches can maximize the preservation of structural information during the SMILES-to-encoding conversion process.

### 3.3. Algorithms of Regression Models

An appropriate machine learning model needs to be employed to identify the underlying relationship between these sub-structural features and the corresponding *k_p_* values. Our approach to predicting the *k*_p_ values of new monomers is based on the statistical correlation between structure and property. We initially explored some complex machine learning algorithms, such as XGBoost (version: 2.0.3) and LightGBM (version: 4.4.0) [25,26], to fit the training data. However, as shown in Figure S6, due to the limited dataset size of only 41 data points, these sophisticated models were unable to effectively learn the inherent relationship between the structures and the *k*_p_ values, resulting in poor fitting performance.

Therefore, it is hypothesized that a simpler multivariate linear regression method may perform better on this small dataset, as it may be able to capture the essential features of the structure–property relationship more robustly. We have compared the performance of several regression methods (Figure S7), including multivariate linear regression [13], Lasso regression [18], ridge regression [13], and Bayesian ridge regression [27].

#### 3.3.1. Multivariate Linear Regression

Multivariate linear regression is the most straightforward and direct method, which can be expressed using the following equation:

(2) $$y=\beta_{0}+\sum_{i=1}^{n}\beta_{i}x_{i}$$

where *y* is the dependent variable, *x* represents the multiple independent variables, and *β*’s are the regression coefficients. Linear regression employs the method of least squares to estimate the values of *β*, thereby minimizing the sum of squared residuals between the predicted and true values. However, the simplicity of linear regression may lead to overfitting, potentially resulting in poor predictive performance on new monomers.

#### 3.3.2. Ridge Regression and Lasso Regression

Ridge regression builds upon the foundation of multivariate linear regression by introducing a regularization penalty term in the loss function to prevent overfitting. The loss function can be expressed as follows:

(3) $$L=\sum {\left(y-X\beta \right)}^{2}+\lambda \sum \beta^{2}$$

where *X* is the feature matrix of independent variables, and the first term $\sum {\left(y-X\beta \right)}^{2\;}$ represents the sum of squared residuals between the predicted and actual values, which quantifies the model’s fit error. The second term $\lambda \sum \beta^{2}$ is the product of the regularization parameter *λ* and the sum of squared regression coefficients. The parameter *λ* can be tuned to control the model complexity, thereby balancing the trade-off between variance and bias. Ridge regression is well suited for situations where there is significant multicollinearity among the predictor variables.

Lasso regression is similar to ridge regression, but the regularization term in the loss function is changed to the sum of the absolute values of the regression coefficients, which can be expressed as the following:

(4) $$L=\sum {\left(y-X\beta \right)}^{2}+\lambda \sum \left|\beta \right|$$

This form of regularization can cause some of the regression coefficients to be precisely shrunk to zero, effectively performing feature selection. Therefore, Lasso regression can automatically select more influential features during the training process and discard the irrelevant ones. Lasso regression ultimately yields a sparse model, which facilitates the interpretation of individual feature influences on the target variable. This renders Lasso regression particularly well suited for scenarios involving a large number of features but a relatively small sample size.

#### 3.3.3. Bayesian Ridge Regression

Bayesian ridge regression is a probabilistic model that extends the concept of ridge regression by incorporating Bayesian principles [27]. It provides a probabilistic approach to estimating regression coefficients, assuming a prior distribution of the coefficients and then deriving their posterior distribution after observing the data. It first assumes that the noise term *β*_0_ in Equation (2) follows a normal distribution with a mean of 0 and a variance of *σ*^2^, while the regression coefficients *β* are assumed to have a Gaussian prior distribution. Subsequently, it is assumed that the noise precision *α*, which is the inverse of *σ*^2^, follows a Gamma prior distribution. Then, using Bayes’ theorem, the posterior distribution of the parameters is calculated based on the prior distributions and the likelihood function of the observed data:

(5) $$p\left(\beta ,\alpha |X,y\right)\propto p\left(y|X,\beta ,\alpha \right)p\left(\beta |\alpha \right)p\left(\alpha \right)$$

where $p\left(y|X,\beta ,\alpha \right)\;$ is the likelihood function of the data, $p\left(\beta |\alpha \right)\;$ is the prior distribution of the coefficients, and $p\left(\alpha \right)$ is the prior distribution of the noise precision. Therefore, Bayesian ridge regression provides a probabilistic interpretation of model parameters, while also enabling automatic feature selection. This imbues the method with the benefits of ridge regression’s suitability for scenarios involving significant multicollinearity among predictor variables, as well as the feature selection capability of Lasso regression.

### 3.4. Validation Methods

Reydt et al. have validated the effectiveness of leave-one-out cross-validation (LOOCV, Figure S8) in the analysis of regression model errors [13]. Therefore, the performance of the models on the training sets was also evaluated using this method. Specifically, multivariate linear regression and Bayesian ridge regression only used a single outer LOOCV, while ridge and Lasso regressions employed both outer and inner LOOCV. The outer LOOCV involved *n* fittings, each time using *n* − 1 monomers as the training data and the remaining one as the test data. The inner LOOCV was performed within the outer LOOCV framework, with *n*(*n* − 1) fittings to determine the optimal regularization parameter *λ*. Ultimately, the n model fittings from the outer LOOCV produced n predictions for each monomer in the training set. The average results of the n models in LOOCV were compared to the experimental values to evaluate the overall fitting performance, thus avoiding the influence of random errors from any single model. Similarly, when predicting *k*_p_ for new monomers, the average prediction of the n models was compared to the experimental data.

## 4. Conclusions

In summary, by using solely the SMILES representations of FRP monomers as input, we have developed a reliable and robust Lasso regression model that provides accurate predictions of *k*_p_ values. This model exhibits strong generalization capabilities, eliminating the need for monomer physical properties during *k*_p_ value predictions. The features in the model provide an accurate description of monomer structural information, enabling reasonable predictions as long as the sub-structural units (atoms, bonds, functional groups) of the new monomers have been encountered in the training set. Attractively, the Lasso regression model achieves a high *R*^2^ of 0.9985 on the training set, and a MAPE of only 5.49% in the predictions for the four new monomers, significantly outperforming the accuracy of quantum chemical calculations as well as previously reported machine learning models. Furthermore, the influence of the ester side chain length of (meth)acrylates on *k*_p_ was accurately predicted by this model, aligning well with established scientific knowledge. This high-accuracy and highly generalizable predictive model allows other researchers to simply input monomer information and rapidly obtain reliable *k*_p_ estimates, thereby accelerating their investigations into FRP mechanisms. In the future, it is worthwhile to explore the extension of this model to other chain-growth systems, such as anionic and cationic polymerization, while ensuring the collection of sufficient kinetic data and the incorporation of solvents and initiators into features.

## Figures and Tables

**Table 1:** Test dataset of *k*_p_ values and Arrhenius parameters for new monomers.

Monomers	Abbr.	*A* [L mol^−1^ s^−1^]	*E*_A_ [KJ mol^−1^]	*k*_p_^25 °C^ [L mol^−1^ s^−1^]
Dodecyl acrylate [19]	DA	10,900,000	15.80	18,588
Tridecyl acrylate [20]	TDA	5,710,000	14.08	19,489
Tert-butyl methacrylate [22]	t-BMA	25,100,000	27.70	352
Chloroprene [21]	CP	19,500,000	26.63	421

**Table 2:** Predicted *k*_p_ values using Lasso regression.

Monomers	*k*_p_ ^25 °C^ [L mol^−1^ s^−1^]
Dodecyl acrylate	17,682
Tridecyl acrylate	20,333
Tetradecyl acrylate	22,034
Pentadecyl acrylate	22,635
Tetradecy methacrylate	611

**Table 3:** Predictive results of Arrhenius parameters and *k*_p_ at multiple temperatures for four new monomers.

Monomers	Abbr.	T [°C]	Predicted *k*_p_ [L mol^−1^ s^−1^]	Predicted *A* [L mol^−1^ s^−1^]	Predicted *E*_A_ [KJ mol^−1^]
Dodecyl acrylate	DA	15	14,621	5,887,746	14.38
Dodecyl acrylate	DA	25	17,682		
Dodecyl acrylate	DA	35	21,396		
Dodecyl acrylate	DA	45	25,672		
Dodecyl acrylate	DA	55	30,192		
Dodecyl acrylate	DA	65	35,402		
Tridecyl acrylate	TDA	15	16,627	7,247,982	14.56
Tridecyl acrylate	TDA	25	20,333		
Tridecyl acrylate	TDA	35	24,705		
Tridecyl acrylate	TDA	45	29,663		
Tridecyl acrylate	TDA	55	34,847		
Tridecyl acrylate	TDA	65	40,749		
Tert-butyl methacrylate	t-BMA	15	270	3,407,435	22.59
Tert-butyl methacrylate	t-BMA	25	381		
Tert-butyl methacrylate	t-BMA	35	505		
Tert-butyl methacrylate	t-BMA	45	664		
Tert-butyl methacrylate	t-BMA	55	857		
Tert-butyl methacrylate	t-BMA	65	1105		
Chloroprene	CP	15	317	4,181,175	22.71
Chloroprene	CP	25	440		
Chloroprene	CP	35	589		
Chloroprene	CP	45	780		
Chloroprene	CP	55	1019		
Chloroprene	CP	65	1284		

## Data Availability

The code in this paper is available at https://github.com/jamesymwang/Kp-predict_MACCS-and-Molecular-Transformer (accessed on 6 September 2024).

## Supplementary Materials

The following supporting information can be downloaded at https://www.mdpi.com/article/10.3390/molecules29194694/s1. Tables S1–S3: Training dataset. Equations (S1)–(S4): Definitions of statistical parameters. Figure S1: Diagram of Molecular Transformer embeddings. Figure S2: Diagram of SMILES processing. Figures S3–S5 and Table S4: Comparison of MACCS fingerprints, Molecular Transformer embeddings, and their combination. Figure S6: Fitting results of XGBoost and LightGBM. Figure S7: Flowchart of algorithms. Figure S8: Diagram of LOOCV. Figure S9: Fitting results at various temperatures. Tables S5–S10: Detailed results for Figure 1, Figure 4 and Figure 5. Reference [28] is cited in the Supplementary Materials.
