# Supplementary material for: A Machine Learning Model for Predicting the Propagation Rate Coefficient in Free-Radical Polymerization
Source: Molecules. 2024 Oct 3;29(19):4694. doi: 10.3390/molecules29194694 (PMC11477705; doi:10.3390/molecules29194694)
Supplement: Supplementary file 1 [file molecules-29-04694-s001.zip › molecules-3220642-supplementary.pdf]

Supplementary Information for
A Machine Learning Model for Predicting the Propagation Rate Coefficient in Free-Radical Polymerization

Yiming Wang, Yue Fang, Haifan Zhou and Hanyu Gao *

Department of Chemical and Biological Engineering, The Hong Kong University of Science and Technology, Hong Kong 999077, China; ywangsx@connect.ust.hk (Y.W.); yfangax@connect.ust.hk (Y.F.); hzhoubz@connect.ust.hk (H.Z.)

* Correspondence: hanyugao@ust.hk

1 Training Dataset

Table S1. FRP Monomers on the training dataset with their abbreviation and canonical SMILES.

Monomers	Abbreviation	Canonical SMILES
n-Butyl acrylate	BA	C=CC(=O)OCCCC
Behenyl acrylate	BeA	C=CC(=O)OCCCCCCCCCCCCCCCCCCCCCC
Benzyl acrylate	BnA	C=CC(=O)OCC1=CC=CC=C1
Heptadecyl acrylate	C17A	C=CC(=O)OCCCCCCCCCCCCCCCCC
Henicosyl acrylate	C21A	C=CC(=O)OCCCCCCCCCCCCCCCCCCCCC
Ethoxyethyl acrylate	EEA	C=CC(=O)OCCOCC
2-ethylhexyl acrylate	EHA	C=CC(=O)OC[C@H](CC)CCCC
Isobornyl acrylate	iBoa	C=CC(=O)O[C@H]1C[C@@H]2CC[C@@]1(C)C2(C)C
Iso-nonyl acrylate	INA-A	C=CC(=O)OCCCCCCC(C)C
Methyl acrylate	MA	C=CC(=O)OC
2-propylheptyl acrylate	PHA	C=CC(=O)OC[C@H](CCC)CCCCC
Stearyl acrylate	SA	C=CC(=O)OCCCCCCCCCCCCCCCCCC
Tert-butyl acrylate	tBA	C=CC(=O)OC(C)(C)C
2-(Hexylcarbamoyloxy)ethyl acrylate	HCEA	C=CC(=O)OCCOC(=O)NCCCCCC
2-(Hexylcarbamoyloxy)isopropyl acrylate	HCPA	C=C(OC(=O)NCCCCCC)C(=O)OC(C)C
Hydroxyethyl methacrylate	HEMA	C=C(C)C(=O)OCCO
2-hydroxypropyl methacrylate	HPMA	C=C(C)C(=O)OC[C@@H](C)O
2-(Phenylcarbamoyloxy)ethyl acrylate	PhCEA	C=CC(=O)OCCOC(=O)NC1=CC=CC=C1
2-(Phenylcarbamoyloxy)isopropyl acrylate	PhCPA	C=C(OC(=O)NC1=CC=CC=C1)C(=O)OC(C)C
Behenyl methacrylate	BeMA	C=C(C)C(=O)OCCCCCCCCCCCCCCCCCCCCCC
Butyl methacrylate	BMA	C=C(C)C(=O)OCCCC
Benzyl methacrylate	BzMA	C=C(C)C(=O)OCC1=CC=CC=C1
Cyclohexyl methacrylate	CHMA	C=C(C)C(=O)OC1CCCCC1
Dodecyl methacrylate	DMA	C=C(C)C(=O)OCCCCCCCCCCCC
2-ethylhexyl methacrylate	EHMA	C=C(C)C(=O)OC[C@H](CC)CCCC
Ethyl methacrylate	EMA	C=C(C)C(=O)OCC
Glycidyl methacrylate	GMA	C=C(C)C(=O)OC[C@@H]1CO1
Iso-butyl methacrylate	iBMA	C=C(C)C(=O)OCC(C)C
Iso-bornyl methacrylate	iBoMA	C=C(C)C(=O)O[C@H]1C[C@@H]2CC[C@@]1(C)C2(C)C
Iso-decyl methacrylate	iDeMA	C=C(C)C(=O)OCCCCCCCC(C)C
Methyl methacrylate	MMA	C=C(C)C(=O)OC
Propylheptyl methacrylate	PHMA	C=C(C)C(=O)O[C@@H](CCC)CCCCCC
n-Pentyl Methacrylate	PnMA	C=C(C)C(=O)OCCCCC
Stearyl methacrylate	SMA	C=C(C)C(=O)OCCCCCCCCCCCCCCCCCC
1,3-Butadiene	BuDE	C=CC=C

Acrylonitrile	CAN	C=CC#N
Methacrylic acid	MAA	C=C(C)C(=O)O
N-vinyl formamide	NVF	C=CNC=O
N-Vinyl Pyrrolidone	NVP	C=CN1CCCC1=O
Styrene	Sty	C=CC1=CC=CC=C1
Vinyl acetate	VAc	C=COC(C)=O

Table S2. Monomer type, k_p values at 25°C and Arrhenius parameters.[1]

Monomers	Type	A [L mol^{-1} s^{-1}]	E_A [KJ mol^{-1}]	$k_p^{25\,°C}$ [L mol^{-1} s^{-1}]
BA	Acrylate	22100000	17.9	16154
BeA	Acrylate	5350000	13.02	28004
BnA	Acrylate	12800000	16.12	19185
C17A	Acrylate	8150000	14.66	22014
C21A	Acrylate	3220000	12.99	17060
EEA	Acrylate	6300000	13.8	24074
EHA	Acrylate	9100000	15.8	15518
iBoa	Acrylate	4810000	15.35	9835
INA-A	Acrylate	13500000	16.54	17080
MA	Acrylate	14100000	17.3	13129
PHA	Acrylate	10500000	16.41	14000
SA	Acrylate	18600000	16.93	20107
tBA	Acrylate	22100000	17.9	16154
HCEA	H-bonding monomers	6600000	13.3	30857
HCPA	H-bonding monomers	6600000	14.1	22346
HEMA	H-bonding monomers	8880000	21.9	1293
HPMA	H-bonding monomers	3510000	20.8	796
PhCEA	H-bonding monomers	12000000	14.3	37479
PhCPA	H-bonding monomers	4900000	14.2	15934
BeMA	Methacrylate	2510000	20.52	638
BMA	Methacrylate	3801894	22.9	370
BzMA	Methacrylate	6760829.8	22.9	657
CHMA	Methacrylate	6309573.4	23	589
DMA	Methacrylate	2511886.4	21	526
EHMA	Methacrylate	2390000	21.6	393
EMA	Methacrylate	4073802.8	23.4	324
GMA	Methacrylate	5011872.3	22.9	487
iBMA	Methacrylate	2640000	21.8	400
iBoMA	Methacrylate	6165950	23.1	553
iDeMA	Methacrylate	2390000	21.6	393
MMA	Methacrylate	2673006.4	22.36	323
PHMA	Methacrylate	2830000	21.72	443
PnMA	Methacrylate	6000000	23.8	406
SMA	Methacrylate	3450000	21.49	593
BuDE	Other	80500000	35.7	45
CAN	Other	1790000	15.4	3587
MAA	Other	380000	16.1	574
NVF	Other	6400000	19.5	2453
NVP	Other	25700000	17.6	21202
Sty	Other	42657952	32.51	86
VAc	Other	13500000	20.4	3599

Table S3. ln(k_p) values of FRP monomers at 15°C，25°C，35°C，45°C，55°C and 65°C.

Monomers	ln(k_p)$^{15°C}$	ln(k_p)$^{25°C}$	ln(k_p)$^{35°C}$	ln(k_p)$^{45°C}$	ln(k_p)$^{55°C}$	ln(k_p)$^{65°C}$
BA	9.439303	9.689908	9.924248	10.143856	10.350080	10.544106
BeA	10.057822	10.240106	10.410559	10.570296	10.720298	10.861428
BnA	9.636175	9.861859	10.072896	10.270666	10.456383	10.631115
C17A	9.794178	9.999422	10.191345	10.371203	10.540099	10.699006
C21A	9.562630	9.744493	9.914553	10.073923	10.223579	10.364384
EEA	9.895689	10.088893	10.269557	10.438864	10.597853	10.747437
EHA	9.428578	9.649782	9.856630	10.050474	10.232504	10.403767
iBoa	8.978839	9.193743	9.394699	9.583022	9.759868	9.926254
INA-A	9.514104	9.745668	9.962203	10.165126	10.355682	10.534967
MA	9.240351	9.482556	9.709040	9.921288	10.120599	10.308122
PHA	9.317054	9.546798	9.761631	9.962959	10.152017	10.329893
SA	9.671782	9.908807	10.130448	10.338156	10.533204	10.716717
tBA	9.439303	9.689908	9.924248	10.143856	10.350080	10.544106
HCEA	10.150918	10.337122	10.511241	10.674413	10.827641	10.971806
HCPA	9.816984	10.014388	10.198979	10.371967	10.534411	10.687248
HEMA	10.331337	10.531541	10.718751	10.894193	11.058941	11.213946
HPMA	9.477408	9.676212	9.862112	10.036327	10.199923	10.353844
PhCEA	6.857854	7.164460	7.451166	7.719849	7.972156	8.209541
PhCPA	6.388829	6.680034	6.952340	7.207527	7.447161	7.672622
BeMA	6.170372	6.457658	6.726298	6.978050	7.214458	7.436884
BMA	5.592134	5.912740	6.212538	6.493489	6.757317	7.005541
BzMA	6.167780	6.488386	6.788184	7.069136	7.332964	7.581188
CHMA	6.056961	6.378967	6.680074	6.962252	7.227232	7.476540
DMA	5.970763	6.264769	6.539692	6.797334	7.039272	7.266901
EHMA	5.670571	5.972977	6.255756	6.520758	6.769609	7.003742
EMA	5.452502	5.780109	6.086452	6.373538	6.643127	6.896770
GMA	5.868444	6.189050	6.488848	6.769800	7.033628	7.281852
iBMA	5.686573	5.991779	6.277176	6.544632	6.795787	7.032088
iBoMA	5.992193	6.315599	6.618015	6.901421	7.167553	7.417945
iDeMA	5.670571	5.972977	6.255756	6.520758	6.769609	7.003742
MMA	5.465244	5.778290	6.071018	6.345345	6.602952	6.845322
PHMA	5.789464	6.093550	6.377900	6.644375	6.894608	7.130042
PnMA	5.672717	6.005924	6.317504	6.609498	6.883694	7.141674
SMA	6.083568	6.384434	6.665773	6.929425	7.177009	7.409949
BuDE	3.301939	3.801749	4.269119	4.707109	5.118404	5.505374
CAN	7.969486	8.185090	8.386701	8.575638	8.753060	8.919988
MAA	6.127494	6.352898	6.563673	6.761198	6.946684	7.121200
NVF	7.532154	7.805159	8.060446	8.299684	8.524341	8.735711
NVP	9.715442	9.961846	10.192259	10.408187	10.610954	10.801729
Sty	3.998459	4.453608	4.879216	5.278069	5.652613	6.005005
VAc	7.902869	8.188475	8.455544	8.705824	8.940850	9.161975

2 Definitions of Statistical Parameters

The equations defining the statistical parameters are as follows:

$$R^2 = 1 - \frac{\sum_{i=1}^{n} (k_{i,exp} - k_{i,pred})^2}{\sum_{i=1}^{n} (k_{i,exp} - \overline{k_{i,exp}})^2} \tag{S1}$$

$$RMSE = \sqrt{\frac{1}{n} \sum_{i=1}^{n} (k_{i,exp} - k_{i,pred})^2} \tag{S2}$$

where n is the dataset size, $k_{i,exp}$ is the experimental ln(k_p), $k_{i,pred}$ is the predicted ln(k_p), $\overline{k_{i,exp}}$ is the average experimental ln(k_p).

$$APE(\%) = \left| \frac{y_{i,exp} - y_{i,pred}}{y_{i,exp}} \right| \times 100\% \tag{S3}$$

$$MAPE(\%) = \frac{1}{n} \sum_{i=1}^{n} \left| \frac{y_{i,exp} - y_{i,pred}}{y_{i,exp}} \right| \times 100\% \tag{S4}$$

Where n is the dataset size, $y_{i,exp}$ is the experimental k_p, A or E_A, $y_{i,pred}$ is the predicted k_p, A or E_A.

3 Definitions of Some Machine Learning Terms

Regularization Parameter: The regularization parameter is a hyperparameter used in machine learning models, particularly in regression and classification algorithms, to prevent overfitting by adding a penalty to the loss function. The penalty discourages the model from fitting too closely to the training data, thus improving its generalization to unseen data. Common types of regularization include L1 (Lasso) and L2 (Ridge) regularization.

Overfitting: Overfitting occurs when a machine learning model learns not only the underlying patterns in the training data but also the noise and outliers. This results in a model that performs very well on the training data but poorly on unseen or test data.

Multicollinearity: Multicollinearity refers to a situation in statistical models, particularly in multiple regression, where two or more predictor variables are highly linearly correlated. This high correlation means that one predictor variable can be linearly predicted from the others with a substantial degree of accuracy. Multicollinearity can lead to several issues such as Unstable Estimates, Inflated Variance, and Reduced Interpretability.

4 Diagram of Molecular Transformer Embeddings and SMILES Processing

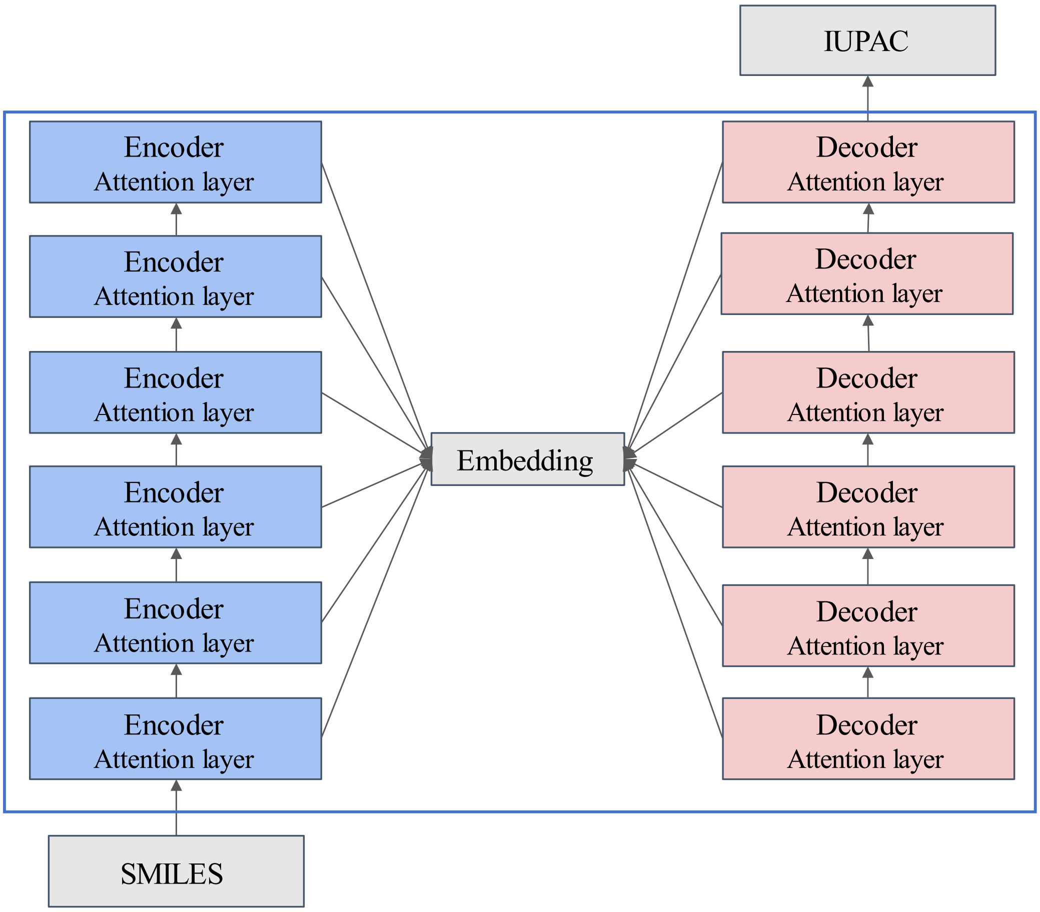

Figure S1. Training process of the Molecular Transformer model. Reprinted with permission from **Ref. (2)**. Copyright 2020 American Chemical Society.[2]

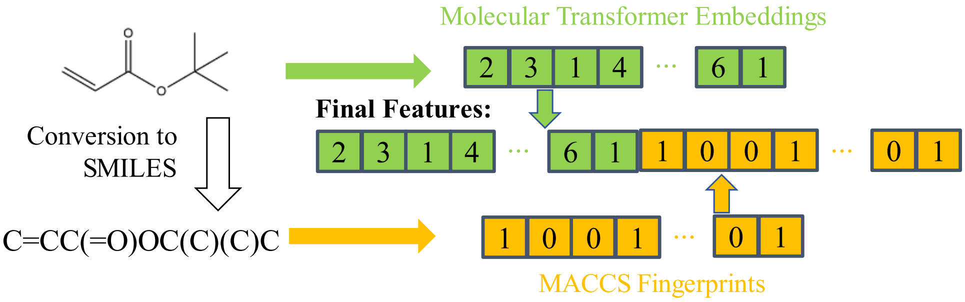

Figure S2. Diagrams of the conversion process of SMILES to final features for regression models.

5 Comparison of MACCS Fingerprints, Molecular Transformer Embeddings, and Their Combination

The SMILES representations[3] of the training set molecules were converted into three distinct input encodings: MACCS fingerprints[4], Molecular Transformer embeddings[2], and their combination. The k_p values at 25°C and a Lasso regression model were then used to compare the performance of these input encodings.

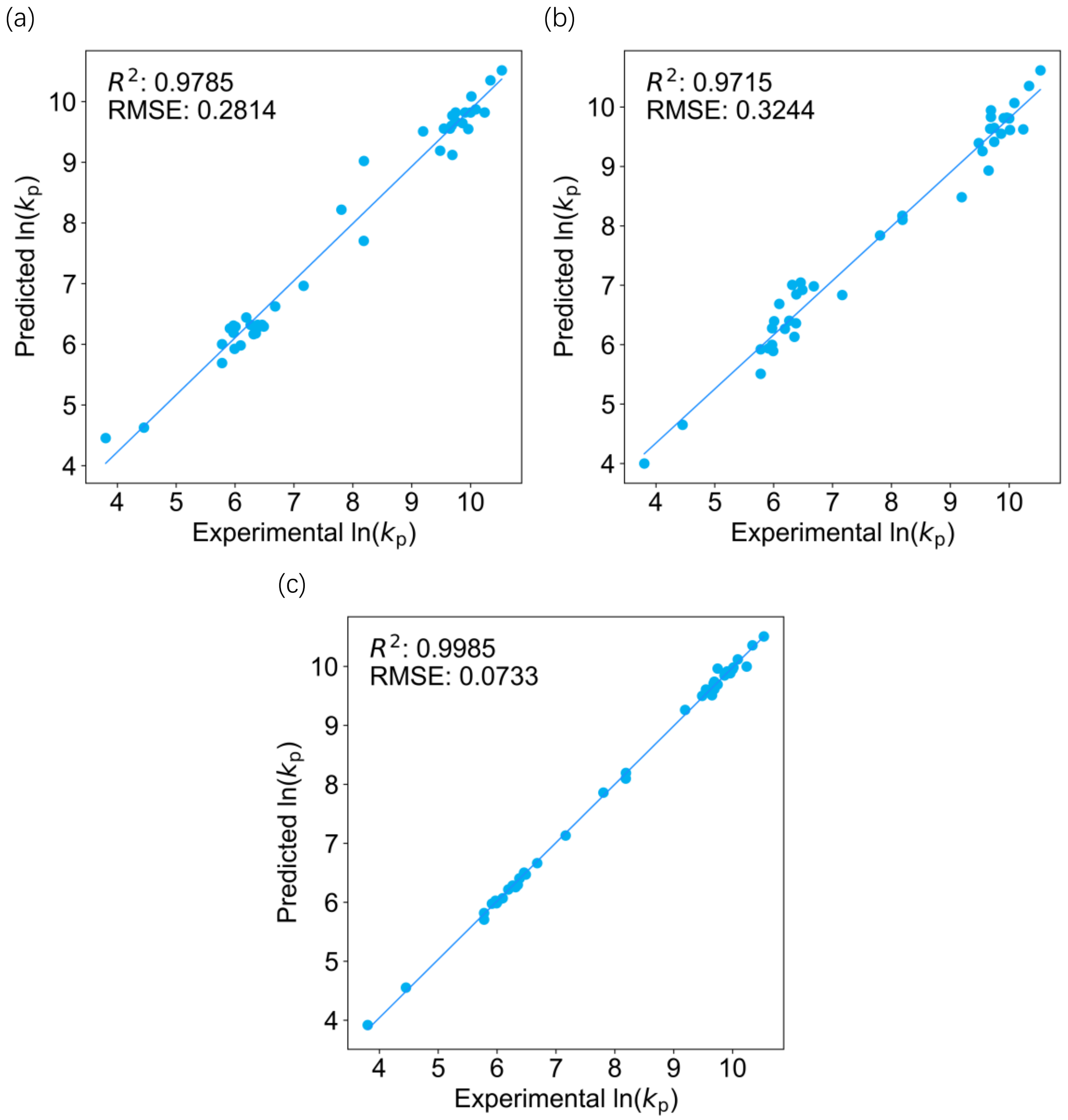

Figure S3. Fitting results on the training dataset for predicting $\ln(k_p)^{25°C}$ versus experimental $\ln(k_p)^{25°C}$: (a) MACCS fingerprints; (b) Molecular Transformer embeddings; (c) Combination.

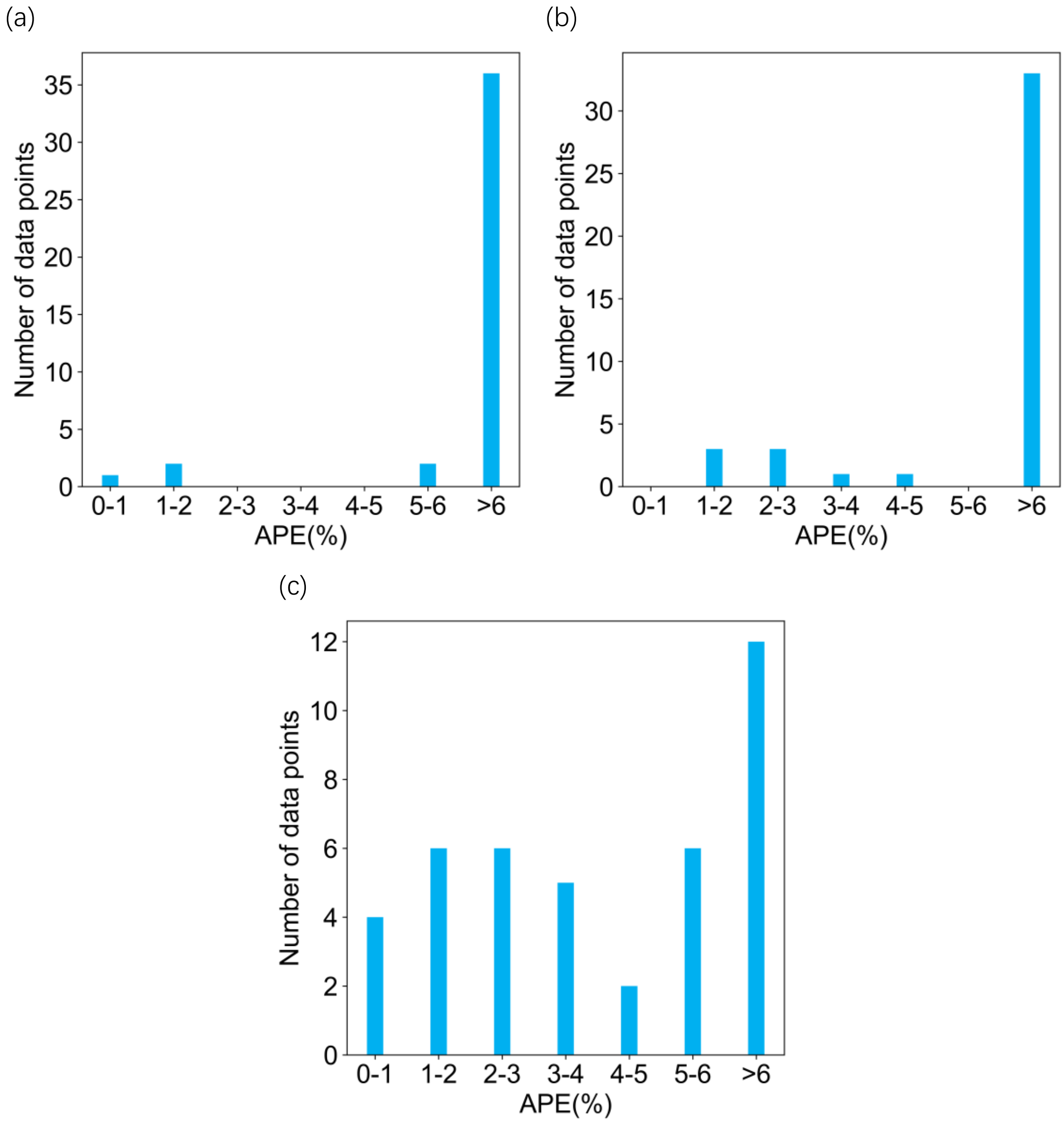

Figure S4. APE distribution of predicted $k_p^{25℃}$ and experimental $k_p^{25℃}$ on the training dataset for (a) MACCS fingerprints; (b) Molecular Transformer embeddings; (c) Combination.

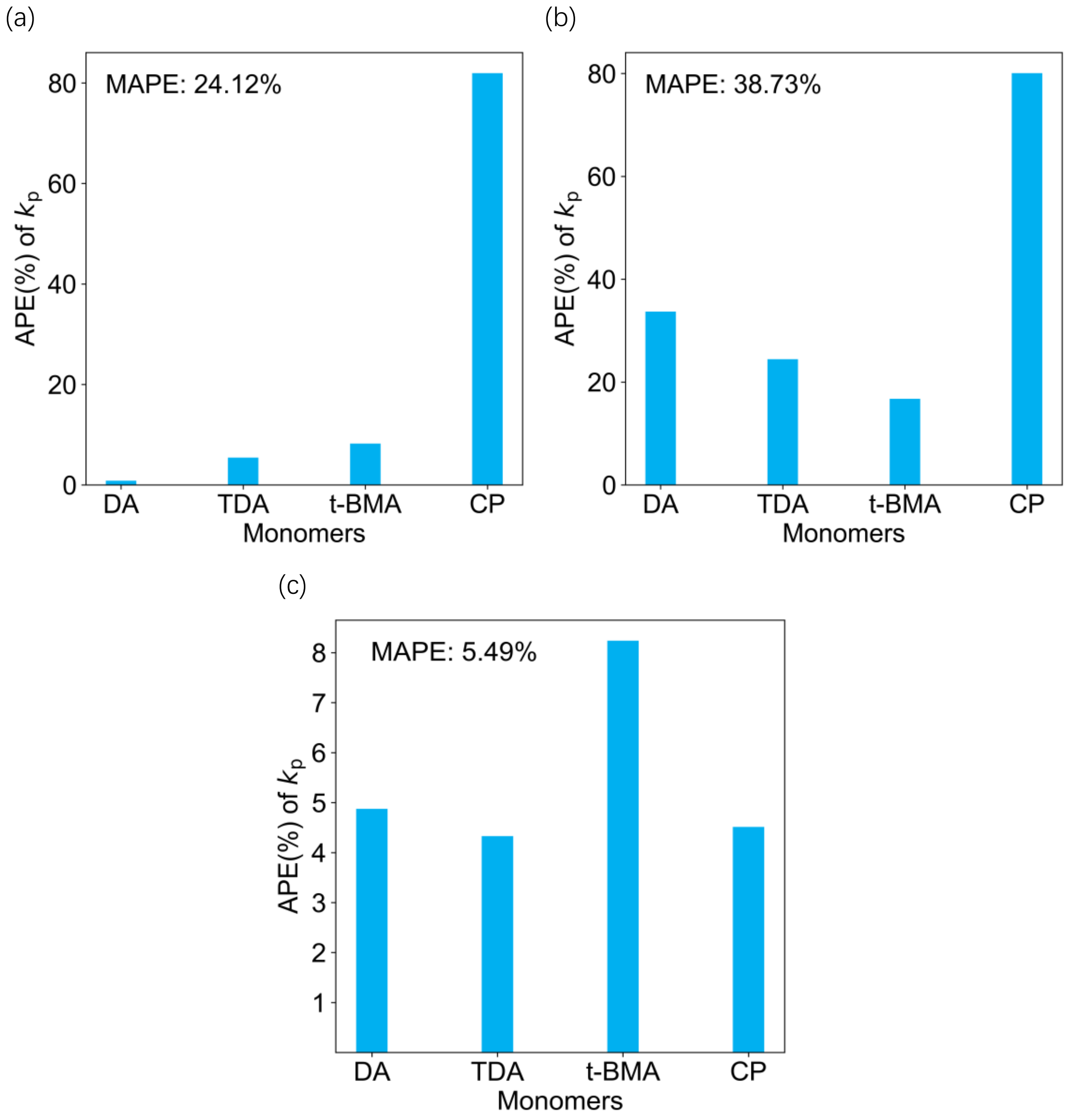

Figure S5. Predictive results on the test dataset: (a) MACCS fingerprints; (b) Molecular Transformer embeddings; (c) Combination.

Table S4. Predictive k_p (L mol^{-1} s^{-1}) at 25°C using different input encodings on the test dataset.

Monomers	MACCS Fingerprints	Molecular Transformer Embeddings	Combination
Dodecyl acrylate	18429	12326	17682
Tridecyl acrylate	18429	14726	20333
Tert-butyl methacrylate	323	293	381
Chloroprene	76	84	440

6 Fitting Results of XGBoost and LightGBM

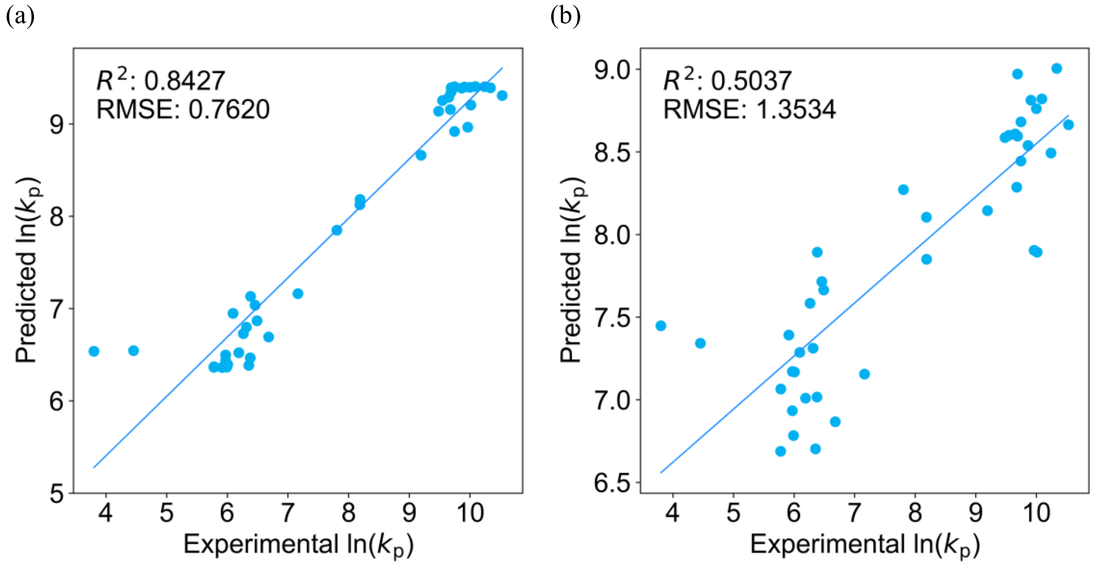

Figure S6. Fitting results on the training dataset for predicting ln(k_p)$^{25°C}$ versus experimental ln(k_p)$^{25°C}$: (a) XGBoost model[5]; (b) LightGBM model.[6]

7 Flowchart of Algorithms

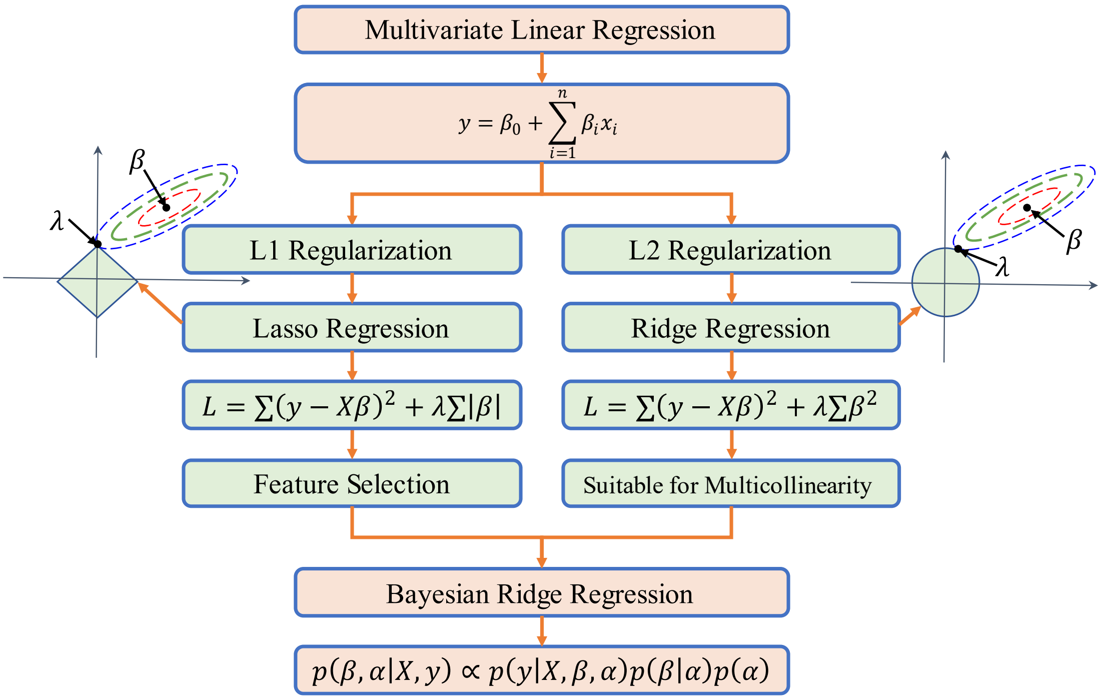

Figure S7. Flowchart of multivariate linear regression, Ridge regression, Lasso regression, and Bayesian ridge regression.

8 Diagram of LOOCV

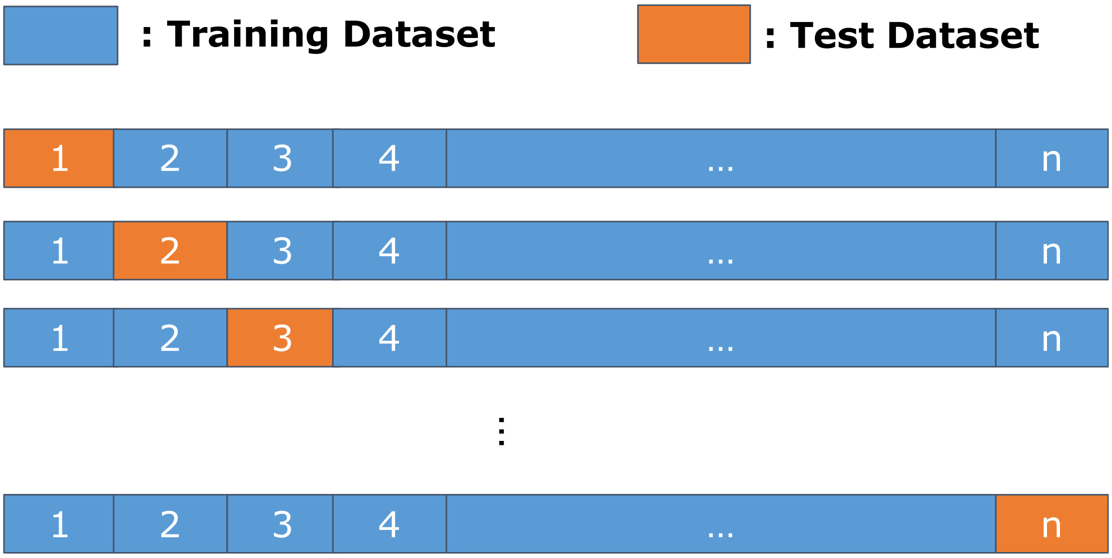

Figure S8. Overview of the leave-one-out cross-validation (LOOCV) strategy. Reprinted from **Ref. (5)**, under CC-BY license.[7]

9 Fitting Results at Various Temperatures

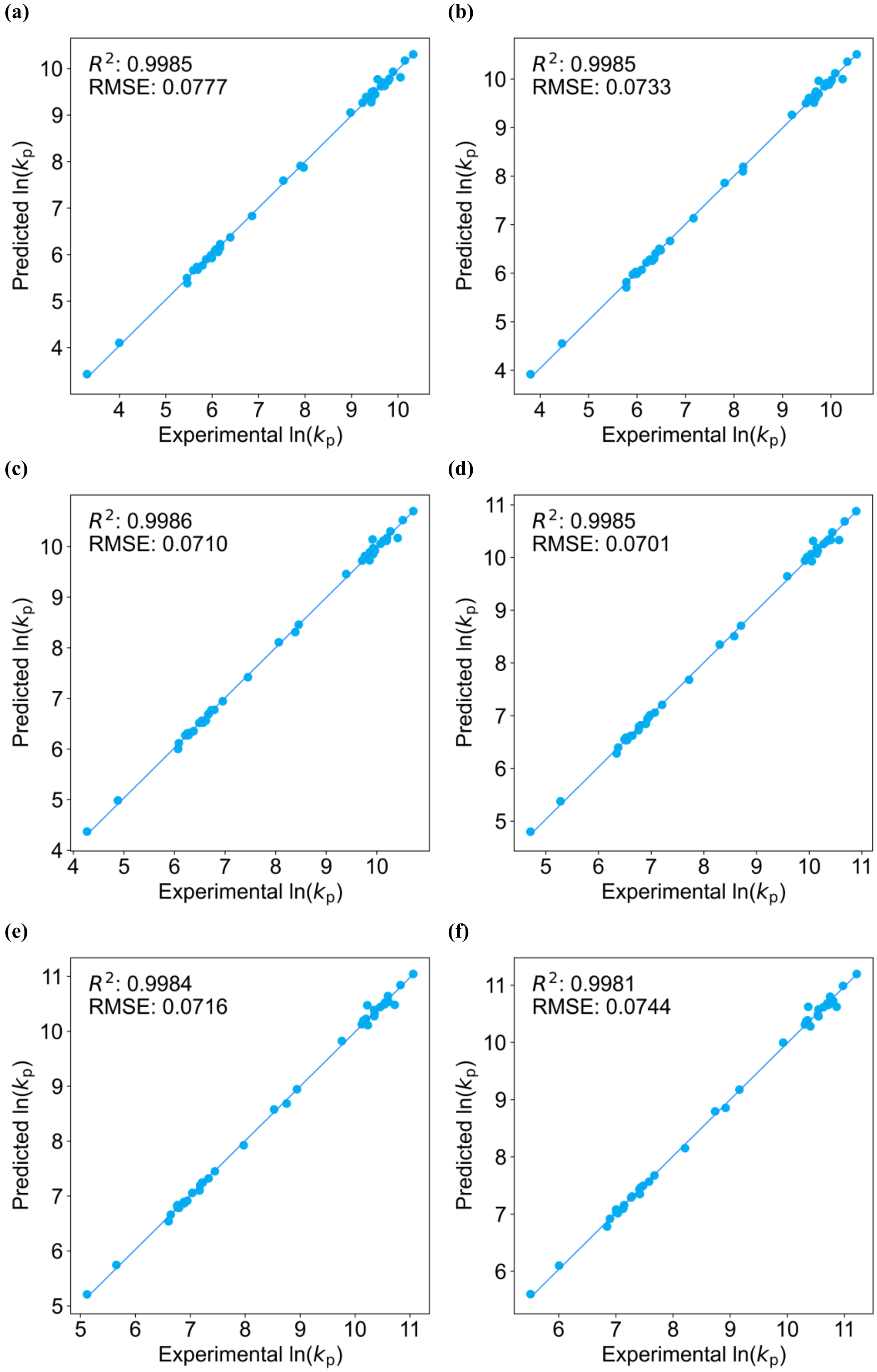

Figure S9. Fitting analyses on the training set using Lasso regression to predict ln(k_p) at: **(a)** 15℃; **(b)** 25℃; **(c)** 35℃; **(d)** 45℃; **(e)** 55℃; **(f)** 65℃.

10 Detailed Results for Figure 1, Figure 4, and Figure 5

Absolute error = |Experimental ln(k_p) -Predicted ln(k_p)|$^{25℃}$

Table S5. Detailed results of multivariate linear regression for Figure 1a.

Monomers	Experimental ln(k_p)$^{25℃}$	Predicted ln(k_p)$^{25℃}$	Absolute error
BA	9.689908	9.675903	0.014005
BeA	10.240106	10.226974	0.013132
BnA	9.861859	9.863718	0.001859
C17A	9.999422	10.010337	0.010915
C21A	9.744493	9.754714	0.010221
EEA	10.088893	10.102922	0.014029
EHA	9.649782	9.636275	0.013507
iBoa	9.193743	9.185776	0.007967
INA-A	9.745668	9.741045	0.004623
MA	9.482556	9.503619	0.021063
PHA	9.546798	9.560402	0.013604
SA	9.908807	9.899476	0.009331
tBA	9.689908	9.685800	0.004107
HCEA	10.337122	10.379516	0.042393
HCPA	10.014388	9.990446	0.023942
HEMA	7.164460	7.124367	0.040093
HPMA	6.680034	6.690005	0.009971
PhCEA	10.531541	10.500998	0.030543
PhCPA	9.676212	9.678212	0.002000
BeMA	6.457658	6.507189	0.049531
BMA	5.912740	5.932040	0.019300
BzMA	6.488386	6.486159	0.002228
CHMA	6.378967	6.429234	0.050267
DMA	6.264769	6.221569	0.043200
EHMA	5.972977	5.976849	0.003872
EMA	5.780109	5.802464	0.022355
GMA	6.189050	6.199556	0.010506
iBMA	5.991779	5.977909	0.013870
iBoMA	6.315599	6.324831	0.009231
iDeMA	5.972977	5.983476	0.010499
MMA	5.778290	5.755760	0.022530
PHMA	6.093550	6.093783	0.000232
PnMA	6.005924	5.986278	0.019646
SMA	6.384434	6.344738	0.039696
BuDE	3.801749	3.844422	0.042673
CAN	8.185090	8.132335	0.052756
MAA	6.352898	6.353613	0.000714

NVF	7.805159	7.890865	0.085706
NVP	9.961846	9.836453	0.125393
Sty	4.453608	4.570228	0.116619
VAc	8.188475	8.188779	0.000303

Table S6. Detailed results of Lasso regression for Figure 1b.

Monomers	Experimental $\ln(k_p)^{25℃}$	Predicted $\ln(k_p)^{25℃}$	Absolute error
BA	9.689908	9.613356	0.076552
BeA	10.240106	9.996747	0.243359
BnA	9.861859	9.845785	0.016074
C17A	9.999422	9.952279	0.047143
C21A	9.744493	9.963030	0.218537
EEA	10.088893	10.119818	0.030924
EHA	9.649782	9.511906	0.137877
iBoa	9.193743	9.262381	0.068638
INA-A	9.745668	9.693064	0.052604
MA	9.482556	9.501159	0.018603
PHA	9.546798	9.609823	0.063025
SA	9.908807	9.916374	0.007567
tBA	9.689908	9.741065	0.051157
HCEA	10.337122	10.358165	0.021043
HCPA	10.014388	9.981991	0.032397
HEMA	7.164460	7.132608	0.031852
HPMA	6.680034	6.665614	0.014420
PhCEA	10.531541	10.509379	0.022162
PhCPA	9.676212	9.709320	0.033108
BeMA	6.457658	6.503506	0.045849
BMA	5.912740	5.976759	0.064019
BzMA	6.488386	6.472403	0.015984
CHMA	6.378967	6.404077	0.025110
DMA	6.264769	6.283593	0.018825
EHMA	5.972977	6.028325	0.055347
EMA	5.780109	5.816753	0.036644
GMA	6.189050	6.217373	0.028323
iBMA	5.991779	5.983831	0.007948
iBoMA	6.315599	6.255933	0.059666
iDeMA	5.972977	5.991025	0.018048
MMA	5.778290	5.705730	0.072560
PHMA	6.093550	6.068651	0.024899
PnMA	6.005924	6.011121	0.005197
SMA	6.384434	6.408221	0.023787
BuDE	3.801749	3.919150	0.117401
CAN	8.185090	8.099401	0.085690
MAA	6.352898	6.295978	0.056920
NVF	7.805159	7.859849	0.054690
NVP	9.961846	9.883339	0.078507
Sty	4.453608	4.552923	0.099315
VAc	8.188475	8.192559	0.004084

Table S7. Detailed results of Ridge regression for Figure 1c.

Monomers	Experimental $\ln(k_p)^{25℃}$	Predicted $\ln(k_p)^{25℃}$	Absolute error
BA	9.689908	9.724569	0.034661
BeA	10.240106	9.940755	0.299351
BnA	9.861859	9.758880	0.102980
C17A	9.999422	9.931771	0.067651
C21A	9.744493	9.940945	0.196452
EEA	10.088893	10.071937	0.016956
EHA	9.649782	9.496537	0.153245
iBoa	9.193743	9.015224	0.178519
INA-A	9.745668	9.715490	0.030179
MA	9.482556	9.535226	0.052670
PHA	9.546798	9.584992	0.038194
SA	9.908807	9.903920	0.004888
tBA	9.689908	9.753081	0.063173
HCEA	10.337122	10.399406	0.062284
HCPA	10.014388	9.933551	0.080837
HEMA	7.164460	7.096382	0.068078
HPMA	6.680034	6.707375	0.027340
PhCEA	10.531541	10.542452	0.010911
PhCPA	9.676212	9.657997	0.018215
BeMA	6.457658	6.581088	0.123430
BMA	5.912740	5.929242	0.016502
BzMA	6.488386	6.564702	0.076316
CHMA	6.378967	6.386502	0.007535
DMA	6.264769	6.260705	0.004064
EHMA	5.972977	6.060323	0.087346
EMA	5.780109	5.846157	0.066048
GMA	6.189050	6.215216	0.026166
iBMA	5.991779	5.916070	0.075709
iBoMA	6.315599	6.489419	0.173820
iDeMA	5.972977	6.007175	0.034198
MMA	5.778290	5.693301	0.084989
PHMA	6.093550	6.098931	0.005381
PnMA	6.005924	5.994455	0.011469
SMA	6.384434	6.464428	0.079994
BuDE	3.801749	3.861572	0.059824
CAN	8.185090	8.148681	0.036410
MAA	6.352898	6.301079	0.051820
NVF	7.805159	7.853714	0.048554
NVP	9.961846	9.917457	0.044390
Sty	4.453608	4.559521	0.105912
VAc	8.188475	8.230445	0.041970

Table S8. Detailed results of Bayesian ridge regression for Figure 1d.

Monomers	Experimental ln(k_p)$^{25℃}$	Predicted ln(k_p)$^{25℃}$	Absolute error
BA	9.689908	9.717059	0.027151
BeA	10.240106	9.958531	0.281575
BnA	9.861859	9.779735	0.082124
C17A	9.999422	9.940624	0.058798
C21A	9.744493	9.952751	0.208257
EEA	10.088893	10.075254	0.013639
EHA	9.649782	9.524869	0.124913
iBoa	9.193743	9.049583	0.144159
INA-A	9.745668	9.724435	0.021233
MA	9.482556	9.528691	0.046135
PHA	9.546798	9.583402	0.036604
SA	9.908807	9.908377	0.000430
tBA	9.689908	9.745498	0.055591
HCEA	10.337122	10.390828	0.053706
HCPA	10.014388	9.947466	0.066921
HEMA	7.164460	7.107391	0.057069
HPMA	6.680034	6.703274	0.023240
PhCEA	10.531541	10.538696	0.007155
PhCPA	9.676212	9.662906	0.013305
BeMA	6.457658	6.561175	0.103517
BMA	5.912740	5.925045	0.012305
BzMA	6.488386	6.550231	0.061845
CHMA	6.378967	6.386598	0.007631
DMA	6.264769	6.260278	0.004490
EHMA	5.972977	6.042217	0.069240
EMA	5.780109	5.835240	0.055132
GMA	6.189050	6.210245	0.021195
iBMA	5.991779	5.929714	0.062065
iBoMA	6.315599	6.457547	0.141948
iDeMA	5.972977	5.999345	0.026368
MMA	5.778290	5.707106	0.071184
PHMA	6.093550	6.094990	0.001440
PnMA	6.005924	5.993179	0.012745
SMA	6.384434	6.437601	0.053167
BuDE	3.801749	3.852909	0.051161
CAN	8.185090	8.152804	0.032287
MAA	6.352898	6.309406	0.043492
NVF	7.805159	7.849366	0.044206
NVP	9.961846	9.920395	0.041451
Sty	4.453608	4.547770	0.094162
VAc	8.188475	8.226094	0.037619

Table S9. Detailed results of 4 models on the test dataset for Figure 4.

Monomers	Experimental $k_p^{25℃}$ [L mol^{-1} s^{-1}]	Multivariate linear regression	Lasso regression	Ridge regression	Bayesian ridge regression
DA[8]	18588	20233	17682	18355	18837
TDA[9]	19489	22838	20333	21521	22151
t-BMA[10]	352	318	381	260	260
CP[11]	421	376	440	75	75

Table S10. Detailed results of E_A and ln*(A)* for Figure 5.

Monomers	Experimental E_A [KJ mol^{-1}]	Predicted E_A [KJ mol^{-1}]	Experimental ln(*A*)	Predicted Ln(*A*)
BA	17.90	17.89	16.911088	16.829268
BeA	13.02	13.12	15.492607	15.289562
BnA	16.12	16.17	16.364956	16.366493
C17A	14.66	15.39	15.913528	16.158985
C21A	12.99	13.81	14.984892	15.533721
EEA	13.80	14.09	15.656060	15.805362
EHA	15.80	16.32	16.023785	16.094363
iBoa	15.35	15.25	15.386208	15.414543
INA-A	16.54	16.85	16.418200	16.490794
MA	17.30	17.01	16.461685	16.364844
PHA	16.41	15.87	16.166886	16.010619
SA	16.93	15.47	16.738672	16.157145
tBA	17.90	17.51	16.911088	16.804660
HCEA	13.30	13.14	15.702580	15.659058
HCPA	14.10	14.12	15.702580	15.678576
HEMA	21.90	21.42	15.999312	15.774933
HPMA	20.80	21.15	15.071127	15.198613
PhCEA	14.30	14.47	16.300417	16.348647
PhCPA	14.20	14.13	15.404746	15.407415
BeMA	20.52	20.12	14.735793	14.623274
BMA	22.90	22.80	15.151010	15.177388
BzMA	22.90	23.00	15.726656	15.749672
CHMA	23.00	22.89	15.657579	15.634661
DMA	21.00	21.09	14.736545	14.789985
EHMA	21.60	21.84	14.686804	14.842996
EMA	23.40	22.97	15.220087	15.083784
GMA	22.90	22.78	15.427320	15.405990
iBMA	21.80	21.71	14.786289	14.742179
iBoMA	23.10	23.03	15.634553	15.546181
iDeMA	21.60	21.59	14.686804	14.701470
MMA	22.36	22.66	14.798714	14.843002
PHMA	21.72	21.70	14.855787	14.822702
PnMA	23.80	23.90	15.607270	15.654907
SMA	21.49	21.34	15.053885	15.018376
BuDE	35.70	35.09	18.203768	18.072965
CAN	15.40	15.89	14.397726	14.508231
MAA	16.10	16.76	12.847927	13.055296
NVF	19.50	19.48	15.671809	15.717321
NVP	17.60	17.76	17.062002	17.044949
Sty	32.51	32.32	17.568724	17.594781
VAc	20.40	20.44	16.418200	16.440017

11 References

(1) Van de Reydt, E.; Marom, N.; Saunderson, J.; Boley, M.; Junkers, T. A Predictive machine-learning model for propagation rate coefficients in radical polymerization. *Polymer Chemistry* **2023**, *14* (14), 1622-1629.

(2) Morris, P.; St. Clair, R.; Hahn, W. E.; Barenholtz, E. Predicting binding from screening assays with transformer network embeddings. *Journal of Chemical Information and Modeling* **2020**, *60* (9), 4191-4199.

(3) Weininger, D. SMILES, a chemical language and information system. 1. Introduction to methodology and encoding rules. *Journal of chemical information and computer sciences* **1988**, *28* (1), 31-36.

(4) Durant, J. L.; Leland, B. A.; Henry, D. R.; Nourse, J. G. Reoptimization of MDL keys for use in drug discovery. *Journal of chemical information and computer sciences* **2002**, *42* (6), 1273-1280.

(5) Chen, T.; Guestrin, C. Xgboost: A scalable tree boosting system. In *Proceedings of the 22nd acm sigkdd international conference on knowledge discovery and data mining*, 2016; pp 785-794.

(6) Ke, G.; Meng, Q.; Finley, T.; Wang, T.; Chen, W.; Ma, W.; Ye, Q.; Liu, T.-Y. Lightgbm: A highly efficient gradient boosting decision tree. *Advances in neural information processing systems* **2017**, *30*.

(7) Cha, G.-W.; Moon, H. J.; Kim, Y.-M.; Hong, W.-H.; Hwang, J.-H.; Park, W.-J.; Kim, Y.-C. Development of a prediction model for demolition waste generation using a random forest algorithm based on small datasets. *International Journal of Environmental Research and Public Health* **2020**, *17* (19), 6997.

(8) Buback, M.; Kurz, C. H.; Schmaltz, C. Pressure dependence of propagation rate coefficients in free-radical homopolymerizations of methyl acrylate and dodecyl acrylate. *Macromolecular Chemistry and Physics* **1998**, *199* (8), 1721-1727.

(9) Haehnel, A. P.; Schneider-Baumann, M.; Arens, L.; Misske, A. M.; Fleischhaker, F.; Barner-Kowollik, C. Global trends for kp? The influence of ester side chain topography in alkyl (meth) acrylates– completing the data base. *Macromolecules* **2014**, *47* (10), 3483-3496.

(10) Pascal, P.; Winnik, M. A.; Napper, D. H.; Gilbert, R. G. Pulsed laser study of the propagation kinetics of tert-butyl methacrylate. *Die Makromolekulare Chemie, Rapid Communications* **1993**, *14* (3), 213-215.

(11) Hutchinson, R.; Aronson, M.; Richards, J. Analysis of pulsed-laser-generated molecular weight distributions for the determination of propagation rate coefficients. *Macromolecules* **1993**, *26* (24), 6410-6415.
